# Studying the Role of Brain Enriched E3 Ubiquitin Ligase TRIM9 in Alzheimer’s Disease

**DOI:** 10.1002/alz.085299

**Published:** 2025-01-03

**Authors:** Anca Gabriela Frasineanu, Stephanie L. Gupton, Stephanie Gupton

**Affiliations:** ^1^ UNC Chapel Hill, Chapel Hill, NC USA

## Abstract

**Background:**

In the last decade, we have demonstrated that the brain‐enriched E3 ubiquitin ligase TRIM9 regulates cytoskeletal dynamics, membrane remodeling, and netrin‐dependent signaling pathways in all stages of neuron development, including the maturation of dendritic spines and electrophysiological activity. Moreover, TRIM9 protein levels increase in the adult brain and are maintained throughout adulthood. In the adult mouse TRIM9 is enriched within the postsynaptic density (PSD), a proteinaceous rich region in the post synapse, containing neurotransmitter receptors, scaffolding proteins, and cytoskeletal elements. The PSD proteome is significantly altered in adolescent mice when TRIM9 is lost, which is linked to changes in synaptic function. Our published proximity labelling experiments to characterize the TRIM9 interactome identified several proteins implicated in synaptic function and Alzheimer’s disease (AD), including Tau.

**Method:**

Cross‐referencing this list with a published dataset of differentially expressed proteins (123) in the hippocampal PSD of 9 month old Tau P301S mice (PS19 AD model) versus non‐transgenic control, revealed a significant percent (56 proteins, 45.5%) overlap. Here we investigate the role of TRIM9 in tau‐mediated neurodegeneration, since TRIM9 is poised to interact with many AD‐associated proteins, and published work indicates that TRIM9 expression in HEK293T cells increased aggregated tau species. Four genotypes were compared (*Trim9^+/+^*, *Trim9^‐/‐^*, *Trim9^+/+^:PS19*, and *Trim9^‐/‐^:PS19*), and 3‐4 animals per genotype and sex were used in each experiment. We analyzed changes in dendritic spine density of six month old murine cortical and hippocampal neurons using the Golgi‐Cox staining method. We also investigated changes in pathological tau accumulation, microglia and astrocytes activation using multiplexed immunofluorescence imaging and biochemical analysis.

**Result:**

Iba1 positive microglia were significantly enriched in the hippocampus and entorhinal cortex of six month old *Trim9^‐/‐^:PS19* mice compared to the other genotypes. Pathological tau accumulation had an increase trend in the hippocampus of *Trim9^‐/‐^:PS19* mice compared to *PS19* only mice.

**Conclusion:**

Deletion of *Trim9* exacerbated the progression of Alzheimer’s disease in six month old *PS19* mice. Future experiments will use primary cultured hippocampal neurons to further determine the mechanism through which *Trim9* deletion increases pathological tau accumulation.

